# Psychosocial working conditions across working life may predict late-life physical function: a follow-up cohort study

**DOI:** 10.1186/s12889-019-7473-y

**Published:** 2019-08-16

**Authors:** Charlotta Nilsen, Ross Andel, Alexander Darin-Mattsson, Ingemar Kåreholt

**Affiliations:** 1 Aging Research Center (ARC), Karolinska Institutet/Stockholm University, Tomtebodavägen 18A, SE-171 65 Stockholm, Sweden; 2 School of Aging Studies, University of South Florida, 13301 Bruce B. Downs Blvd, MHC 1323, Tampa, Florida, 33612 USA; 3 The International Clinical Research Center, St. Anne’s University Hospital, Brno, Czech Republic; 4 Department of Neurology, 2nd Faculty of Medicine, Charles University and Motol University Hospital, Prague, Czech Republic; 5 Institute of Gerontology, School of Health and Welfare, Aging Research Network – Jönköping (ARN-J), Jönköping University, Box 1026, 55111 Jönköping, Sweden

**Keywords:** Work-related stress, Job control, Mobility limitations, Life course, Cohort, Sweden

## Abstract

**Background:**

Increasing life expectancy has made understanding the mechanisms underlying late-life health and function more important. We set out to investigate whether trajectories of change in psychosocial working conditions are associated with late-life physical function.

**Methods:**

Two Swedish surveys, linked at the individual level, were used (*n* = 803). A psychosocial job exposure matrix was used to measure psychosocial working conditions during people’s first occupation, as well as their occupation every five years thereafter until baseline in 1991. Physical function was measured in 2014. Random effects growth curve models were used to calculate intraindividual trajectories of working conditions. Predictors of physical function were assessed with ordered logistic regression.

**Results:**

A more active job at baseline was associated with increased odds of late-life physical function (OR 1.15, CI 1.01–1.32). Higher baseline job strain was associated with decreased odds of late-life physical function (OR 0.75, CI 0.59–0.96). A high initial level followed by an upward trajectory of job strain throughout working life was associated with decreased odds of late-life physical function (OR 0.32, CI 0.17–0.58).

**Conclusions:**

Promoting a healthier workplace by reducing chronic stress and inducing intellectual stimulation, control, and personal growth may contribute to better late-life physical function.

## Background

The demographic transition to an aging society comes with challenges. As we age, disease and disability tend to increase [1, 2], negatively impacting individuals and placing more pressure on health and social care systems [3]. Limitations in physical function have been associated with lower independence and lower health-related quality of life [4, 5], as well as higher likelihood of multimorbidity and severe disability [1, 2]. Disability in old age is associated with the need for home help service use and institutional care [3]. Because of the importance of late-life health and physical function to older adults and society, it is crucial to identify factors that influence these outcomes.

We spend a large portion of our adult lives at work, and working conditions may play an important role in shaping health in late-life health (e.g., [6–10]). Work-related stress is one of the major sources of stress in adulthood. Chronic stress at work predicts ill health during working life, including sleep disturbance, musculoskeletal pain, coronary heart disease, depressive symptoms, anxiety, fatigue, emotional exhaustion, and diabetes [11–16]. There is also growing evidence of long-term associations that reach far into life after retirement, and a few studies have found that work-related stress predicts functional impairment in old age [7, 10, 17–20]. However, most earlier studies have been restricted to measuring working conditions at one or two points in time and may thus have overlooked the health-defining role of people’s careers, which typically span decades [21].

One way to understand long-term associations between stress and late-life physical function is the concept “allostatic load,” a term for the accumulated wear and tear on the body that results from chronic stress [22]. Stress is an acute response to an acute situation, during which our body turns on an instinctual physiological fight-or-flight response. Although this response can be lifesaving under truly life-threatening conditions, it is less helpful when triggered by psychological stress. When the stress response becomes chronic, the wear-and-tear aftereffects of the response are magnified [22].

This physiological stress reaction may have long-term consequences for physical function via physiological dysregulations that disturbs hormone production, metabolism, immune function, and blood pressure regulation and causes damage to brain, muscles, heart, and vascular system [23]. The physical frailty that follows aging may further exacerbate these adverse processes [24–26]. Moreover, some consequences may not occur until much later in life because exposures accumulate over the life course. For instance, work life trajectories characterized by stress combined with an unhealthy lifestyle can cause risks to accumulate [24, 27]. Examples include stress combined with physical inactivity or stress combined with smoking; both smoking and physical inactivity predict limitations in physical function later in life [28].

One of the most well-established models for measuring psychosocial work environment is the job demand-control model, originally constructed by Karasek [29]. This environmentally based model measures stressors in the work environment (“work stressors”) and categorizes jobs by control and demand: active jobs are those with high psychological demands and high control, passive jobs are those with low psychological demands and low control, high strain jobs are those with high psychological demands and low control, and low strain jobs are those with low psychological demands and high control.

Two hypotheses have been derived from this model: the job strain hypothesis and the active learning hypothesis. According to the job strain hypothesis, having high job strain is the most stressful situation. According to the active learning hypothesis, active jobs promote active learning, and high control counteracts the stress of high demands, making the job less stressful. With time, accumulated learning experiences may facilitate feelings of confidence and mastery, which may encourage an active life outside work [30]. In fact, those in active jobs are the most active during their leisure time, both during working life [31–35] and after retirement [36]. Remaining active as one ages; for example, through physical activity, may preserve or enhance physical function later in life [28].

The overall aim of the study was to investigate long-term associations between psychosocial working conditions throughout working life and late-life physical function. To better understand how the relationship between psychosocial working conditions and late-life physical function is shaped, in this national representative study, we included psychosocial working conditions at up to eight points in time. This enabled us to capture different trajectories of high strain jobs and active jobs during working life and investigate their relationships with late-life physical function.

## Methods

### Data and analytic sample

Data were derived from two Swedish surveys with individual-level information: the Level of Living Survey (LNU) 1991 [37] and the Swedish Panel Study of Living Conditions of the Oldest Old (SWEOLD) 2014 [38]. LNU 1991 was based on a random sample of the Swedish population from 18 through 75 years. People in the LNU sample who had reached at least 76 years were included in SWEOLD. Data were collected through face-to-face interviews. To enable us to link 1991 LNU data on paid employment with data on physical function from SWEOLD 2014, the participants had to be between 47 and 71 years in 1991. The response rate of people between those ages in LNU 1991 (*n* = 2183) was 75.5%.

A total of 1243 were between the ages of 47 and 71 in 1991 with a paid employment, 368 participants died before the follow-up in 2014, and 70 did not respond for other reasons. Missing baseline data (0.2%) reduced the study sample to 803 participants, and item nonresponse at follow-up reduced it further, to 801 (417 women and 384 men) born between 1920 and 1944.

SWEOLD 2014 data were mainly collected through computer-assisted telephone interviews of people 70 years and older. Mixed interviews (0.5%) and proxy interviews (4.6%) were used when needed to avoid non-response due to impaired cognition or poor health. Proxies were typically a spouse, close relative, or health care professional. Postal questionnaires were used as a final alternative (10.7%).

### Variables

*Physical function*, the dependent variable, was assessed as the self-reported ability to stand without support (yes/no), walk up and down stairs without difficulty (yes/no), walk 100 m fairly briskly without difficulty (yes/no), and get up from a kitchen chair without using the support of your arms (yes/no), as well as self-reported problems with balance indoors (yes/no). The answers were combined into an index ranging 0 to 5, where 5 indicated the greatest physical function. Because few people scored 0, 1, or 2, these categories were combined.

*Psychosocial working conditions* were assessed on the basis of self-reported main occupation in 1991 and self-reported occupations during working life. The occupations were coded using a psychosocial job exposure matrix (JEM) [39, 40]. Occupational history was assessed with retrospective questions about the respondent’s first occupation that lasted at least six months and all occupations held by the respondent one by one in temporal order thereafter [41]. First occupation and occupation at age 25, 30, 35, 40, 45, and 50 (based on occupational history), and occupation in 1991 were then matched with the JEM. Both the matrix and the occupation variables in LNU were coded using the 1980 Nordic version of the three-digit International Standard Occupational Classification Codes. The JEM was created on the basis of a random sample of 12,084 Swedish workers from the 1977 and 1979 Swedish Survey of Living Conditions (ULF). The ULF surveys contained items specific to the job demand-control model. To create the matrix, average scores that ranged from 0 to 10 for job demands and control were generated separately for women and men for 262 occupations. The occupation-based job control scale consisted of a linear composite of 12 items: influence over the planning of work, setting of work pace, how time is used in work, selection of supervisor, selection of co-workers, planning of work breaks, planning of vacations, flexible work hours, varied task content, varied work procedures, opportunity to learn new things, and experience of personal fulfillment on the job. The job demand scale consisted of a linear composite of 2 items constructed on the basis of responses to two items, i.e., psychologically demanding (taxing) work and hectic work.

In our analytic sample, occupation-based psychological job demand scores in 1991 ranged from 1.3 to 8.2 (mean = 4.9, SD = 1.4), and occupation-based job control scores from 1.8 to 8.2 (mean = 5.3, SD = 1.2). Job variables were created for each person’s first occupation and their occupation every five years thereafter until baseline in 1991. Demands and control were measured on continuous scales to best capture the variability in how active the active jobs were and how stressful the high strain jobs were. Jobs with a “demand” score above 5 (range 0–10) were considered high demands and jobs with a “control” score above 5 (range 0–10), high control.

**Fig. 1:**
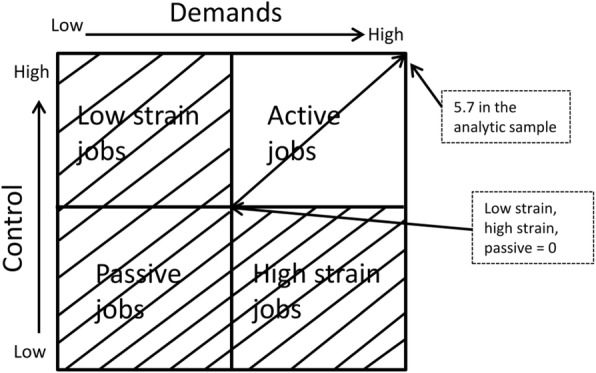
The construct of the active job variable. Source: Adapted from Karasek & Theorell (1990) and modified by authors

*Active jobs*. We first re-scaled the demands and control variables by subtracting 5 and coding values below 0 as 0. That is, scores of 5 or lower on the job demands and job control variable were coded as 0. Second, we summed the transformed demands and control variables to create the active job variable, in which jobs with demands and/or control values of 5 or below were classified as non-active and all other jobs as active. Non-active jobs included high strain jobs (those with high demands and *low* control), low strain jobs (those with *low* demands and high control), and passive jobs (those with *low* demands and *low* control). High strain jobs, low strain jobs, and passive jobs all had a score of 0 in this variable, and all scores above 0 indicated some level of active jobs (5.7 indicated the most active jobs in our analytic sample). See Fig. 1.

**Fig. 2:**
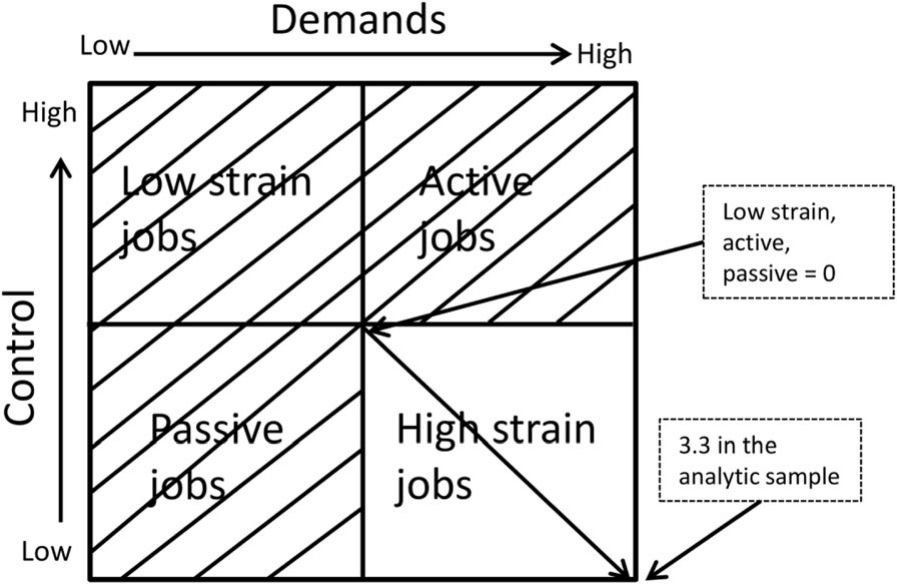
The construct of the high strain job variable. Source: Adapted from Karasek & Theorell (1990) and modified by authors

*High strain jobs*. The original control variable was reversed so that a score of 10 indicated the lowest level of control and 0 the highest level of control. In a next step, the demand and control variables were re-scaled by subtracting 5 and coding values below 0 as 0. That is, scores of 5 or lower on the job demands and/or job control variable (i.e., *low* job demands or *high* job control) were coded as 0. The re-scaled demands and control variables were then summed to create the high strain job variable. Non-high strain jobs (i.e., low strain jobs, active jobs, and passive jobs) all had a score of 0 in this variable, and all scores above 0 indicated some level of job strain (3.3 indicated the highest degree of job strain in our analytic sample). See Fig. 2.

Data on occupational history and occupation at baseline were used to construct *trajectories of active jobs* and *trajectories of high strain jobs*. Random effects growth curve models were used to calculate within-person change. Random effects allow for variation between participants in the individual intercept and slope [42]. The intercept of the trajectories was divided in low and high via a median split. The slope was divided into downward (−), stable (0), and upward (+) slope groups. A slope was considered upward if there was an increase of more than a half standard deviation above zero and downward if there was a decrease below zero. The intercept and slope of the trajectories were then combined to create a variable with five categories, all within the same quadrant: low starting point and downward slope (*low/−*), low starting point and stable slope (*low/0*), low starting point and upward slope (*low/+*), high starting point and downward slope (*high/−*), high starting point and stable (*high/0*), and high starting point and upward slope (*high/+*). Theoretically there should have been six categories, but no participants fell in the category *low/−.* The other four categories were then compared to the category *low/0*.

### Statistical analyses

All statistical analyses were conducted with STATA 15. After testing the assumption of proportional odds, ordered logistic regression was used to analyze late-life physical function. The only statistically significant difference between women and men found was among people who started their working life lower in the active job quadrant and experienced an upward trajectory. These results are presented in the text and not in a table. Because we found no other statistically significant interactions between sex and psychosocial working conditions (with *p* < 0.05) in their association with late-life physical function, we analyzed women and men together, controlling for sex.

Prior to giving control variables linear representation, we first tested linearity via dummy representation. All analyses were adjusted for age (linear representation), sex, and a 4-item index of physical working conditions (heavy lifting, exposure to vibrations, daily sweating, and other physically demanding work) that ranged from 0 to 4 (linear representation). *Model II* was further adjusted for baseline level of education assessed across four categories: compulsory, vocational, upper secondary, and university (linear representation) and occupation-based social class divided in two categories: blue-collar workers (including small farmers and entrepreneurs without employees) and white-collar workers (including farmers and entrepreneurs with employees and academic professionals). *Model III* was further adjusted for physical activity and smoking at baseline. Physical activity (linear representation) was assessed with one question at baseline and at follow-up: “Do you engage in any exercise, outdoor, or sporting activity, such as long walks, and how often?” Response alternatives were 0 = no; 1 = yes, but rarely; 2 = yes, one to three times per month; 3 = yes, approximately once a week; and 4 = yes, several times a week. Smoking was assessed with the question “Do you smoke?” Response alternatives were dichotomized into no (“no, never”) and yes (“yes” or “yes, but I have quit”).

## Results

In the analytic sample, physical function decreased with age. Also, people who were blue-collar workers, had lower levels of education, were less physically active, smoked, or had worse physical working conditions had worse late-life physical function (Table 1).

**Table 1:** Descriptive statistics at baseline: covariates

	Analytic sample n (%)	Mean late-life physical function (range 0–3)
All		2.3
Age at baseline (mean 54)		
*47–55*	541 (67.4)	2.5
*56–65*	246 (30.6)	1.9
*66–71*	16 (2.0)	1.4
Sex		
*Women*	417 (51.9)	2.3
*Men*	386 (48.1)	2.3
Physical working conditions^a^		
*0*	408 (50.8)	2.4
*1*	206 (25.7)	2.3
*2*	126 (15.7)	2.1
*3*	47 (5.9)	2.0
*4*	16 (2.0)	2.6
Level of education		
*Compulsory*	249 (31.0)	2.1
*Vocational*	384 (47.8)	2.3
*Upper secondary*	93 (11.6)	2.4
*University*	77 (9.6)	2.6
Occupation-based social class		
*Blue-collar worker*	306 (38.1)	2.2
*White-collar worker*	497 (61.9)	2.4
Smoking		
*No, never*	370 (46.1)	2.4
*Yes or yes but I have quit*	433 (53.9)	2.2
Physical activity		
*No*	99 (12.3)	2.0
*Yes, but rarely*	60 (7.5)	2.1
*Yes, 1–3 times/month*	56 (7.0)	2.1
*Yes, once a week*	224 (27.9)	2.4
*Yes, several times a week*	364 (45.3)	2.4

^a^A 4-item index of physical working conditions (heavy lifting, exposure to vibrations, daily sweating, and other physically demanding work) that ranged from 0 to 4

People in the active job quadrant who experienced an upward trajectory had better late-life physical function than people who experienced other trajectories. People who started their working life lower in the high-strain quadrant and experienced a stable trajectory had better late-life physical function than those who experienced other trajectories (Table 2).

**Table 2:** Descriptive statistics at baseline: psychosocial working conditions

	Analytic sample n (%)	Mean late-life physical function (range 0–3)
All		2.3
Active jobs in 1991^a^	300 (37.4)	2.5
Trajectories *Low/0*	279 (34.7)	2.4
*Low/+*	75 (9.3)	2.5
*High/−*	35 (4.4)	2.3
*High/0*	202 (25.2)	1.8
*High/+*	212 (26.4)	2.5
High strain jobs 1991^b^	82 (10.2)	2.0
Trajectories *Low/0*	397 (49.4)	2.4
*Low/+*	11 (1.4)	1.8
*High/−*	106 (13.2)	1.9
*High/0*	243 (30.3)	1.9
*High/+*	46 (5.7)	1.8

Low/0 = low starting point and stable slope, Low/+ = low starting point and upward slope, High/− = high starting point and downward slope, High/0 = high starting point and stable slope, High/+ = high starting point and upward slope

^a^Based on a dichotomized active job variable in which all people with a value > 0 in the active job variable were grouped together. High strain jobs, low strain jobs, and passive jobs are non-active jobs. ^b^Based on a dichotomized high strain variable

People who had a more active job at baseline in 1991 had increased odds of late-life physical function (Odds Ratio (OR) 1.15, Confidence Interval (CI) 1.01–1.32) in the fully adjusted model (Table 3, model 3). That is, each step higher on the active job scale increased the odds of having one more physical function by 15%. No statistically significant associations were found between trajectories in the active job quadrant and late-life physical function. However, in the active job quadrant, among those who had a low starting point and an upward trajectory, we observed a difference between women and men. An interaction between trajectories in this quadrant and sex (Model 3, *p* = 0.03) indicated that a low starting point and an upward trajectory was associated with increased odds of late-life physical function in women (OR 2.13, CI 0.93–6.89) and decreased odds in men (OR 0.86, CI 0.33–2.22).

**Table 3:** Associations between psychosocial working conditions and late-life physical function

	Late-life physical function		
	Model 1	Model 2	Model 3
Active job quadrant	OR (95% CI)	OR (95% CI)	OR (95% CI)
*Active job in 1991*^a^	**1.20 (1.06,1.37)**	**1.19 (1.03,1.35)**	**1.15 (1.01,1.32)**
*Trajectories*	*Ref. = low/0*	*Ref. = low/0*	*Ref. = low/0*
Low/+	1.28 (0.74,2.22)	1.32 (0.73,2.38)	1.54 (0.85,2.78)
High/−	0.87 (0.43,1.75)	0.76 (0.37,1.56)	0.68 (0.33,1.43)
High/0	0.79 (0.51,1.23)	0.77 (0.49,1.19)	0.78 (0.50,1.22)
High/+	1.45 (0.92,2.27)	1.28 (0.78,2.13)	1.20 (0.73,2.00)
High strain quadrant			
*High strain job in 1991*^b^	0.82 (0.65,1.04)	0.79 (0.62,1.01)	**0.75 (0.59,0.96)**
*Trajectories*	*Ref. = low/0*	*Ref. = low/0*	*Ref. = low/0*
Low/+	0.79 (0.23,2.70)	0.69 (0.20,2.33)	0.48 (0.14,1.64)
High/−	**0.64 (0.41,0.98)**	**0.64 (0.41,0.99)**	0.68 (0.44,1.05)
High/0	0.85 (0.61,1.19)	0.79 (0.56,1.11)	0.81 (0.58,1.15)
High/+	**0.34 (0.19,0.61)**	**0.34 (0.18,0.61)**	**0.32 (0.17,0.58)**

Results in bold: *p* value <0.05. Low/0 = low starting point and stable slope, Low/+ = low starting point and upward slope, High/− = high starting point and downward slope, High/0 = high starting point and stable slope, High/+ = high starting point and upward slope. All analyses were adjusted for age, sex, and physical working conditions at baseline (1991). Model 2 was further adjusted for level of education and occupation-based social class at baseline. Model 3 was further adjusted for smoking and physical activity at baseline. ^a^The scale ranged from 0 to 5.7 and was given linear representation. High strain jobs, low strain jobs, and passive jobs have a score of 0 in this scale. ^b^The scale ranged from 0 to 3.3 and was given linear representation. Low strain jobs, active jobs, and passive jobs have a score of 0 in this scale

Higher job strain at baseline in 1991 was associated with decreased odds of late-life physical function (OR 0.75, CI 0.59–0.96) in the fully adjusted model (Table 3, model 3). All trajectories in the high strain quadrant were associated with decreased odds of late-life physical function. However, this association was not always statistically significant. A high starting point and downward trajectory was associated with decreased odds of late-life physical function (OR 0.64, CI 0.41–0.99) after adjusting for age, sex, physical working conditions, social class, and level of education (reference group: a low starting point and a stable trajectory [low/0]) (Table 3, model 2). After further adjusting for physical activity and smoking at baseline, this association was statistically nonsignificant (Table 3, model 3). We also observed a relatively strong association between a high starting point and upward trajectory and decreased odds of late-life physical function (OR 0.32, CI 0.17–0.58) in the fully adjusted model (reference group: low/0) (Table 3, model 3). No statistically significant (*p* < 0.05) differences were found between women and men in the associations between high strain jobs and late-life physical function.

## Discussion

This study investigated whether psychosocial working conditions are related to late-life physical function. To the best of our knowledge, no previous studies have investigated the relationship between late-life physical function and trajectories of psychosocial working conditions measured over a period of time as long as that examined in this study. The main results can be summarized as follows: Those with a more active job at baseline had better late-life physical function (i.e., 70 years and older). Those with greater job strain at baseline had worse late-life physical function. Starting working life with a high strain job and experiencing a trajectory of increasing strain over working life was particularly strongly associated with limitation in late-life physical function.

Previous studies have observed long-term associations between work-related stress in midlife and limitations in late-life physical function [7, 10, 17–20]. However, none of those studies investigated trajectories of psychosocial working conditions. People’s careers – that is, their jobs over the course of their working life – may have a crucial impact on health [21]. It is therefore important to investigate psychosocial working conditions over time rather than only at a single point in time. The results of the present study suggest that it is harmful to remain in a stressful occupation in which the stress increases over time, and that it is important to find strategies early in the career to prevent such a pathway. People who started their working life in the upper part of the high strain quadrant and experienced a downward trajectory had decreased odds of late-life physical function. Perhaps these individuals, after a long period of job strain, changed their occupation, either voluntarily or as a result of illness due to chronic stress. In 2015, the World Health Organization (WHO) released the *World report on ageing and health* [43], in which the authors emphasize that a life-course approach is crucial to better understand the factors that contribute to healthy aging. Our findings underscore existing findings that work-related stress throughout working life is important to health both before and after retirement. However, more research is needed to fully understand the impact of work-related stress on late-life health and function.

In people in the active job quadrant, there was a pattern whereby those with a stable or downward trajectory experienced more limitations in late-life physical function than those with an upward trajectory. However, those results were not statistically significant. Only the most recent active job reported (job in 1991) was statistically significantly associated with higher odds of late-life physical function. This may indicate that active jobs later in the career matter more for late-life function than accumulating a history of active jobs over the course of working life; i.e., that it is the experience closest to retirement that we carry over into life after retirement.

There are several possible explanations for why active jobs may be related to better late-life physical function. Studies show that having an active job encourages an active lifestyle outside work [31, 33–36] and that an active lifestyle earlier in life predicts later-life engagement in leisure activities, such as physical activity [44]. Also, active jobs seem to be replaced by a physically, socially, and intellectually active lifestyle after retirement [36]. Regardless of the origin of active leisure after retirement, remaining physically active helps preserve and may even enhance late-life physical function [28]. Another possible explanation for the association between active jobs and better late-life physical function may be the relationship between late-life cognitive and physical function [45]. Both intellectually stimulating jobs and active leisure in midlife have independently been associated with better late-life cognitive function [46, 47], which in turn may be associated with better late-life physical function. Moreover, as suggested by the results of the present study and earlier research [7, 9, 10, 17–20], having a stressful job in midlife is associated with limitations in late-life physical and cognitive function. Hence, having an active job, in which high control counteracts the stress of high demands, may diminish the negative influence of stress on the body in later life.

### Limitations and strengths

The analytic sample in this study was based on a national random sample with high response rates at both baseline and follow-up. The work history provided in LNU 1991 gave us a unique opportunity to investigate trajectories of psychosocial working conditions throughout working life. Using an average population-based matrix does not take into account interindividual variation in stress stemming from the same occupation. Still, it has the advantage of being relatively free of bias caused by individual reporting differences and is internally valid [40, 48].

As in earlier studies [7, 18, 19], this study showed that neither an unhealthy lifestyle nor socioeconomic factors earlier in life had much impact on the association between psychosocial working conditions and late-life physical function. Although several confounders were accounted for in the analyses, it is still possible that we did not completely eliminate all confounders, such as other unfavorable aspects of socioeconomic factors and lifestyle factors. Moreover, unlike psychosocial working conditions, lifestyle and socioeconomic factors were only measured at baseline and not over the life course. Also, we did not have the possibility to investigate change in late-life physical function.

We also need to be aware of selective survival when conducting studies that include the oldest old: our results may underestimate the associations between psychosocial working conditions and physical function in old age, as the people with poor health may have died before follow-up.

The use of postal questionnaires as a final alternative may have led to an underestimation of morbidity in the oldest old, as non-response in old age is likely to be related to poorer health [49]. However, the inclusion of proxy interviews results in a better representation of the older population [50]. Finally, few people in the high job strain quadrant began their working life in the lower part of the quadrant and then experienced an upward trajectory. This may have increased the risk of type II error.

### Potential implications

Because the older population is growing rapidly, it is vital to identify modifiable factors that may delay or prevent the onset of disability. Consistent with prior research, the present study found that psychosocial working conditions play a role in shaping late-life health and function. The workplace could therefore serve as an arena for such preventive strategies. The results of this study suggest that it is important to find strategies to reduce stress at work as early as possible in people’s careers. Over time, reinforcing employees’ control over decision-making and the development and use of their skills at work (i.e., inducing job control) may reduce the perception of situations as stressful and induce intellectual stimulation and personal growth, as long as the psychological job demands are equal to the person’s capabilities [51]. Autonomy and empowerment, employee involvement, and individual development and growth are important factors that may contribute to a healthy workplace, together with skilled communication; accessible, positive, and fair leadership; appropriate staffing; collaboration/teamwork; and safe physical work [52].

## Conclusions

To conclude, promoting a healthier workplace by reducing chronic stress and inducing intellectual stimulation, control, and personal growth, may contribute to better late-life physical function. In turn, such initiatives may not only improve the health of workers but may also lower the cost of health and social care by improving health and function of the older population. Hence, investing in a healthy workplace could be a double win for society.

## Data Availability

The datasets used and/or analyzed during the current study are available from the corresponding author on reasonable request. Public access to the database may be given if applicants have a scientific affiliation, sign a statement that the data will only be used for scientific purposes, and that the scientific project have ethical approval. Applicable sections of The Swedish Research Council (VR) principles for conducting research in humanities and the social sciences must be adhered to (http://www.codex.vr.se/en/forskninghumsam.shtml). Data will be available after the above-mentioned documents have been received, by e-mail at the SWEOLD Research Data Center dataaccess@sweold.se More information can be found at www.sweold.se

## Abbreviations

CI: Confidence Interval
LNU: the Level of Living Survey
OR: Odds Ratio
SWEOLD: the Swedish Panel Study of Living Conditions of the Oldest Old
ULF: the Swedish Survey of Living Conditions
WHO: the World Health Organization
